# Inconsistent condom use among Ugandan university students from a gender perspective: a cross-sectional study

**DOI:** 10.3402/gha.v7.22942

**Published:** 2014-04-10

**Authors:** Devika Mehra, Per-Olof Östergren, Björn Ekman, Anette Agardh

**Affiliations:** Division of Social Medicine and Global Health, Department of Clinical Sciences, Lund University, Malmö, Sweden

**Keywords:** condom efficacy, gender, peer norms, Uganda, condom use, HIV

## Abstract

**Background:**

Feminization of the HIV/AIDS epidemic has been a prominent phenomenon in sub-Saharan Africa. Inconsistent condom use among young people is one of the major risk factors in the continued propagation of the epidemic. Therefore, it is of importance to increase knowledge of gender aspects of condom use among young people.

**Objective:**

To investigate whether gender differences regarding individual and social factors determine the association between condom efficacy and inconsistent condom use with a new sex partner, among Ugandan university students.

**Design:**

In 2010, 1954 Ugandan students participated in a cross-sectional survey, conducted at Mbarara University of Science and Technology in southwestern Uganda. A self-administered questionnaire assessed socio-demographic factors, alcohol consumption, sexual behaviors (including condom use and condom efficacy), and peer norms. The data were stratified by sex and examined by multivariate logistic regression analysis.

**Results:**

A total of 1,179 (60.3%) students reported having had their sexual debut. Of these, 231 (37.4%) males and 209 (49.2%) females reported inconsistent condom use with a new sex partner. Students with low condom efficacy had a higher risk of inconsistent condom use with a new sex partner, even after adjusting for the potential confounders. A synergistic effect was observed between being a female and low condom efficacy with inconsistent condom use.

**Conclusion:**

The association between inconsistent condom use and low condom efficacy was found among both males and females, but females were found to be at a higher risk of inconsistent condom use compared to their male counterparts. Therefore, gender power relations should be addressed in policies and interventions aiming at increasing condom use among young people in sub-Saharan settings. Programs could be designed with intervention strategies that focus on interactive and participatory educational activities and youth-friendly counseling of young people, which in turn may improve their interpersonal communication and condom negotiation skills with their partners.

The feminization of the HIV/AIDS epidemic has been a prominent phenomenon in sub-Saharan Africa (1). Individuals between the ages of 15 and 24 have been severely affected, and 75% of all new HIV cases in this age group occur in women (2). In sub-Saharan Africa, the epidemic has increased due to socio-cultural factors, behind which are gender inequalities (3). Uganda has one of the world's youngest populations, with a high national prevalence of HIV (7.3%) (4). The prevalence in the age group cited above differs from males (2.1%) to females (4.9%). Furthermore, a greater increase was seen in females (7.1%) than in males (2.8%) between the ages of 20 and 24 years (4).

Risky sexual behaviors among young people in Uganda have been measured in a recent survey that showed 24% had more than one sexual partner during the past 12 months, 79% had a non-regular partner, and 10% had been affected by STI (sexually transmitted infections)-related symptoms (5). This population is therefore at high risk for STIs and unintended pregnancies. Results from studies targeting university students in Uganda showed a higher risk of inconsistent condom use among female students, in comparison to their male counterparts (5, 6).

Condom use among young people is determined by individual and social factors. Perceived self-efficacy is one of the individual factors that can influence condom use. It is a concept derived from social cognitive theory and is considered as a factor that could potentially lead to health-related behavioral change (7). Perceived self-efficacy is defined as confidence in one's ability to exhibit motivation and capability to achieve a given goal (8). Condom efficacy is a person's confidence in his or her ability to successfully use a condom during sexual intercourse (9). Such efficacy requires risk reduction and self-regulation skills, but possessing the skills and being able to transform them into action under difficult circumstances are two different matters (10).

In the Ugandan context, where gendered cultural norms and inequitable power relations prevail, women have less control in a sexual relationship (11). Interpersonal communication along with behavioral skills between partners is an integral part of a relationship that determines behavior (12, 13). A positive attitude towards condoms and a greater confidence in one's ability to use them consistently in various circumstances corresponds to higher levels of condom use (14, 15).

There are various factors that influence condom use. In prior studies ‘intention to use a condom’ has been shown to be an important predictor (16, 17). The theory of planned behavior (18) and its extended versions (19) have suggested that in the absence of environmental barriers, any behavior is more likely to occur if there is a strong intention and ability to carry it out. Behavioral intentions in turn are determined by attitudes, subjective norms, and self-efficacy which account for considerable variation in actual condom use in different situations (18).

Individual factors affecting behavior change should not be seen in isolation. Numerous social factors influence the behaviors of young people. Peer norms can exert considerable pressure on young people and affect their decisions (20). According to studies conducted among US university students, discussions about romantic relationships, alcohol consumption, and sexual behaviors can shape perceptions of what their peers consider normative behavior (21, 22). Normative perceptions of sexual experiences can be an important influence in student's decision to engage in risky sexual activities such as having multiple sexual partners, using condoms inconsistently, and alcohol consumption in conjunction to sex (22). Studies conducted among Ethiopian, Cambodian, and Laotian adolescents have supported the notion that peer influences can affect risky sexual practices (23, 24). Another study in South Africa of young people, showed that higher self-efficacy to communicate with peers, increased the likelihood of condom use (25). The same study showed that more females communicated with their peers than did their male counterparts.

Consistent condom use is determined by a number of factors, some of which are linked to gender in more or less obvious ways. Some of these factors prevent inconsistent condom use, while others may work in the opposite direction. Knowledge of whether these factors contribute to feminization of the HIV/AIDS epidemic in sub-Saharan Africa appears to be incomplete. Although some African studies have documented the association between self-efficacy and condom use (25, 26), gender differences are not very well examined. Therefore, the aim of this study is to investigate whether gender differences regarding individual and social factors (peer norms) determine inconsistent condom use with a new sexual partner, particularly with regard to condom efficacy among university students in Uganda.

## Methods

### Study design and setting

The study was conducted at the Mbarara University of Science and Technology (MUST), a public institution that is the second largest university in Uganda. It is located in the center of Mbarara, approximately 350 km to the southwest of the capital city, Kampala. In 2010, the number of universities in Uganda expanded resulting in 29 new institutions of higher learning. A greater number of students are now being enrolled in universities than in the past. Those students receiving government scholarships live on campus during their entire course of study, while others remain on campus during their first and second years and then move on to privately run hostels.

We analyzed a cross-sectional data set of undergraduate students from the university's four faculties: science, medicine, computer science, and development studies. The sample consisted of 1,954 participating students out of a total enrolment of 2,706, representing 72% of all undergraduates. As the outcome of the study was risky sexual behavior, the analysis was based on a subset of 1,179 students who stated that they had debuted sexually. Of the respondents, 58.8% were male (*n*=693) and 41.2% female (*n*=486). The Institutional Review Committee at MUST granted ethics approval for the project.

### Data collection and analysis

The data were collected by means of an 11-page self-administered questionnaire with 132 questions based on socio-demographic factors, academic progress, social capital, mental health, sexual behavior (condom efficacy and condom use), alcohol consumption, and other lifestyle variables. The questionnaire was also used in previous studies of university students in this setting (6, 27, 28).

The entire undergraduate student body at MUST was invited to take part in the survey. Prior to the questionnaire distribution, a consent form was circulated describing the purpose of the study, and students were asked to sign if they agreed to participate. The research team informed the students that participation in the survey was voluntary and anonymity would be assured. The contact details of the project's principal investigator and the research assistant were provided in case students had any personal questions. The signed consent forms, and completed questionnaires were deposited by each student in a sealed box.

### Definition of variables

#### Background variables

*Socio-demographic variables (individual level)*. Age was categorized as ≤23 (‘younger’) and >23 (‘older’). The cut-off was based on the median age of our study sample.

Area of growing up was dichotomized into rural or urban. The latter option combined peri-urban and small town.

Educational level of head of the household during childhood was categorized as ‘did not complete primary school’ or ‘completed primary school’, which were coded as ‘≤primary school’ and ‘>primary school’.

The role of religion in the family while growing up was dichotomized into major role (‘religion played a big role or was relatively important’) or minor role (‘religion was not so important or not important at all’).

#### Sexual behavior variables

Pleasure of using a condom was based on the question ‘How do you compare the degree of pleasure using a condom during intercourse with not using one?’. The responses were ‘no difference’ and ‘more pleasure with a condom’ and coded as: ‘same or more pleasure’ and ‘less pleasure’ remained coded as ‘less pleasure’.

Intention to use a condom was based on the statement ‘I intend to use a condom whenever I have intercourse with a new sex partner’ Those who responded were coded as ‘yes’ and others were classified as ‘no’.

Multiple sexual partners was determined by the response to the question ‘How many sexual partners have you had during the last 12 months?’ and was dichotomized into ‘0 to 1’ and ‘≥2’. The respondents in the latter category were classified as having multiple sexual partners.

#### Exposure variable

Condom efficacy was constructed by combining two statements: ‘I am satisfied with my ability to use a condom correctly’ and ‘I believe I can persuade a new sex partner to use a condom’. The respondents who indicated ‘yes’ regarding both statements were coded as ‘high efficacy’ and the other responses were categorized as ‘low efficacy’.

*Social level (peer norms)*. Peers using a condom with a new sex partner was based on the statement ‘My friends at the university always use a condom with a new partner’. The response alternatives were ‘yes’ or ‘no’.

Peers having difficulty demanding condom use was based on the statement ‘My friends at the university have difficulty demanding condom use with a new partner’. The response alternatives were ‘yes’ or ‘no’.

Alcohol consumption on the occasion of sexual intercourse had the following response alternatives: ‘always or almost always’, ‘more often than on half of the occasions’, ‘about half of the occasions’, ‘more seldom than a quarter of the occasions’ and ‘almost never or never’. The first three options were coded as ‘frequent user’ and the last two as ‘infrequent user’.

#### Dependent variable

Inconsistent condom use was ascertained by asking the question ‘How often do you use a condom with a new sexual partner?’ The response option ‘always’ was coded as ‘consistent condom use’ and the other alternatives (‘often’, ‘sometimes’, ‘never’, ‘does not apply to me’) were coded as ‘inconsistent condom use’.

### Statistical analysis

Statistical analysis was conducted using PASW (SPSS) statistical package Version 21.0. We first measured the prevalence of the variables we used within our sample population. Logistic regression analysis then calculated the crude odds ratio (OR) with 95% confidence interval (CI) for investigating the association between socio-demographic factors, condom efficacy, intention to use a condom, pleasure of using a condom, alcohol consumption in relation to sex, and peer norms in conjunction with inconsistent condom use with a new sexual partner. Multivariate logistic regression was used step-wise to control for the potential confounders of age, area of origin, pleasure of using a condom, intention to use a condom, multiple sexual partners, alcohol in relation to sexual intercourse and peer norms. Estimates of effect modification (synergy) were done as ‘departure from additivity of effects on the chosen outcome scale’ and calculation of synergy index (SI) was carried out to disclose effect modification between the chosen variables as proposed by Rothman and Greenland (29).

The following algorithm was used, whereby SI>1 signifies a synergistic effect (representing a positive effect modification) and SI<1 an antagonistic effect (representing a negative effect modification):

$$\text{SI}=\frac{\left(\text{O}{\text{R}}_{\left(1+1\right)}-1\right)}{\left(\text{O}{\text{R}}_{\left(1+0\right)}-1)+(\text{O}{\text{R}}_{\left(0+1\right)}-1\right)}$$

where: OR_(1+1)_=odds ratio for dummy variable exposed to both factors

OR_(1+0)_=odds ratio for dummy variable exposed to one factor

OR_(0+1)_=odds ratio for dummy variable exposed to other factor

OR_(0+0)_=odds ratio for the dummy variable unexposed to both factors

Significant effect moderation between two variables indicates that when both are present there is an amplified effect, i.e. the combination of the two indicators has a stronger effect, which is higher than added effect of the variables in question.

## Results

Table 1 gives the prevalence of all the socio-demographic factors, condom efficacy, intention to use a condom, pleasure of using a condom, peer norms, and other sexual behavior variables of the total sample. The stratification of the sample was done on the basis of sex.

**Table 1:** Prevalence of socio-demographic factors, alcohol consumption, sexual behaviors (including condom efficacy), and condom use among Ugandan university students

		All *n*=1,179 (%)	Male *n*=693 (%)	Female *n*=486 (%)	*X* ^2^ p*
Individual level					
Age	≤23	743 (65.0)	407 (60.5)	336 (71.5)	0.001
	>23	400 (35.0)	266 (39.5)	134 (28.5)	
	Missing	(36)	(20)	(16)	
Area of growing up	Urban	607 (51.7)	336 (48.8)	271 (56.0)	0.015
	Rural	566 (48.3)	353 (51.2)	213 (44.0)	
	Missing	(6)	(4)	(2)	
Educational level of	>Primary	820 (71.0)	465 (68.6)	355 (74.4)	0.018
head of household	≤Primary	335 (29.0)	213 (31.4)	122 (25.6)	
	Missing	(24)	(15)	(9)	
Religion	Major role	731 (62.5)	400 (58.2)	331 (68.5)	0.001
	Minor role	439 (37.5)	287 (41.8)	152 (31.5)	
	Missing	(9)	(6)	(3)	
Condom efficacy	High	764 (75.4)	485 (77.8)	279 (71.5)	0.025
	Low	249 (24.6)	138 (22.2)	111 (28.5)	
	Missing	(166)	(70)	(96)	
Intention to use a condom	Yes	393 (33.3)	267 (38.5)	126 (25.9)	0.001
with any new sex partner	No	786 (66.7)	426 (61.5)	360 (74.1)	
Pleasure using a condom	No difference/More pleasure	319 (33.2)	175 (30.0)	144 (38.2)	0.009
	Less pleasure	641 (66.8)	408 (70.0)	233 (61.8)	
	Missing	(219)	(110)	(109)	
Condom use with a new partner	Consistent	603 (57.8)	387 (62.6)	216 (50.8)	0.001
	Inconsistent	440 (42.2)	231 (37.4)	209 (49.2)	
	Missing	(136)	(75)	(61)	
Sexual partners in the last 12 months	0–1	680 (66.4)	356 (58.7)	324 (77.5)	0.001
	≥2	344 (33.6)	250 (41.3)	94 (22.5)	
	Missing	(155)	(87)	(68)	
In a relationship	Yes	923 (80.2)	519 (77.0)	404 (84.7)	0.001
	No	228 (19.8)	155 (23.0)	73 (15.3)	
	Missing	(28)	(19)	(56)	
Alcohol on the occasion	Infrequent users	391 (79.0)	245 (76.6)	146 (83.4)	0.084
of sexual intercourse^a^	Frequent users	104 (21.0)	75 (23.4)	29 (16.6)	
	Missing	(95)	(68)	(27)	
Social Level (peer norms)					
Friends always use condom	Yes	660 (67.4)	400 (66.8)	260 (68.4)	0.625
with new partner	No	319 (32.6)	199 (33.2)	120 (31.6)	
	Missing	(200)	(94)	(106)	
Friends have difficulty demanding	Yes	501 (50.7)	311 (51.4)	190 (49.5)	0.558
condom use with new partner	No	488 (49.3)	294 (48.6)	194 (50.5)	
	Missing	(190)	(88)	(102)	

* *p*-value in table is analyzed based on sex.

a Analyzed only for those who consumed alcohol.

A higher percentage of females in our sample (71.5%) were younger than 23 years compared to their male counterparts (60.5%). A greater proportion of females (28.5%) reported low condom efficacy than males (22.2%). Intention to use a condom with a new sex partner was higher in females (74.1%) than in males (61.5%). A larger majority of male respondents (70%) reported less pleasure with a condom as compared to females (61.8%). Inconsistent condom use with a new sexual partner was higher among females (49.2%) than males (37.4%). Approximately two thirds of the students (67.4) reported that their friends use a condom with a new sex partner; there was not much difference between males and females.

Table 2 provides an analysis of the associations between socio-demographic factors and condom efficacy in relation to inconsistent condom use with a new sex partner. Growing up in a rural environment was significantly associated with inconsistent condom use. This association was significant for males (OR crude 1.81, 95% CI 1.30–2.52) and females (OR crude 1.67, 95% CI 1.13–2.47). Our main exposure, low condom efficacy, had a significant association with inconsistent condom use, for males (OR crude 4.66, 95% CI 3.08–7.07) and females (OR crude 6.45, 95% CI 3.84–10.81). Intention to use a condom with a new sex partner did not show an association (OR crude 0.98, 95% CI 0.76–1.27), with no significant results found in males and females. Less pleasure using a condom was significantly associated (OR crude 1.43, 95% CI 1.07–1.90), with no gender difference in males and females. Multiple sexual partners in the last 12 months had a significant negative association with inconsistent condom use among males (OR crude 0.69, 95% CI 0.48–0.97), and the point estimate was similar for females (OR crude 0.70, 95% CI 0.43–1.11). Frequent consumption of alcohol on the occasion of sexual intercourse showed a significant association (OR crude 1.64, 95% CI 1.05–2.57), but there was no obvious gender difference. The response that friends do not always use a condom with a new partner was associated with the outcome among males (OR crude 1.48, 95% CI 1.03–2.13) and females (OR crude 1.81, 95% CI 1.14–2.87). Reporting that friends would have difficulty demanding that a condom be used with a new sex partner was associated with inconsistent condom use (OR crude 1.40, 95% CI 1.06–1.82) but our study found no gender differences in this regard.

**Table 2:** Association between socio-demographic factors, alcohol consumption, sexual behavior, condom efficacy, and inconsistent condom use among Ugandan university students

		All *n* (%)	OR (95% CI)	Male *n* (%)	OR (95% CI)	Female *n* (%)	OR (95% CI)
Individual level							
Sex	Male	231 (37.4)	1 (Ref)				
	Female	209 (49.2)	**1.62 (1.26–2.08)**				
Age	≤23	138 (38.9)	1 (Ref)	79 (33.1)	1 (Ref)	59 (50.9)	1 (Ref)
	>23	285 (43.5)	1.21 (0.93–1.57)	145 (40.1)	1.35 (0.96–1.90)	140 (47.6)	0.88 (0.57–1.35)
Area of growing up	Urban	200 (36.4)	1 (Ref)	94 (30.5)	1 (Ref)	106 (43.8)	1 (Ref)
	Rural	239 (48.9)	**1.67 (1.30–2.14)**	136 (44.3)	**1.81 (1.30–2.52)**	103 (56.6)	**1.67 (1.13–2.47)**
Educational level of head of	>Primary	298 (41.0)	1 (Ref)	148 (36.2)	1 (Ref)	150 (47.3)	1 (Ref)
household	≤Primary	136 (45.9)	1.22 (0.93–1.60)	80 (40.8)	1.22 (0.86–1.72)	56 (56.0)	1.42 (0.90–2.23)
Religion	Major role	272 (42.4)	1 (Ref)	126 (35.8)	1 (Ref)	146 (50.3)	1 (Ref)
	Minor role	164 (41.7)	0.97 (0.75–1.26)	102 (39.2)	0.86 (0.57–1.30)	62 (46.6)	1.16 (0.83–1.61)
Condom efficacy	High efficacy	206 (28.9)	1 (Ref)	125 (27.7)	1 (Ref)	81 (30.9)	1 (Ref)
	Low efficacy	157 (68.6)	**5.38 (3.90–7.42)**	82 (64.1)	**4.66 (3.08–7.07)**	75 (74.3)	**6.45 (3.84–10.81)**
Intention to use condom	No	283 (42.4)	1 (Ref)	129 (35.6)	1 (Ref)	154 (50.3)	1 (Ref)
with new partner	Yes	157 (41.9)	0.98 (0.76–1.27)	102 (39.8)	1.20 (0.86–1.66)	55 (46.2)	0.85 (0.55–1.30)
Pleasure using a condom	No difference/More pleasure	103 (34.3)	1 (Ref)	51 (30.7)	1 (Ref)	52 (38.8)	1 (Ref)
	Less pleasure	255 (42.8)	**1.43 (1.07–1.90)**	148 (39.3)	1.46 (0.99–2.15)	107 (48.9)	1.51 (0.97–2.33)
Sexual partners in	0–1	281 (45.4)	1 (Ref)	132 (41.0)	1 (Ref)	149 (50.2)	1 (Ref)
the last 12 months	≥2	115 (34.6)	**0.64 (0.48–0.84)**	78 (32.2)	**0.69 (0.48–0.97)**	37 (41.1)	0.70 (0.43–1.11)
In a relationship	Yes	358 (42.5)	1 (Ref)	179 (37.5)	1 (Ref)	179 (49.0)	1 (Ref)
	No	71 (39.7)	0.89 (0.64–1.23)	45 (36.3)	0.95 (0.63–1.43)	26 (47.3)	0.93 (0.53–1.64)
Alcohol on the occasion of	Infrequent user	143 (38.3)	1 (Ref)	82 (35.0)	1 (Ref)	61 (43.9)	1 (Ref)
sexual intercourse^a^	Frequent user	49 (50.5)	**1.64 (1.05–2.57)**	34 (47.2)	1.66 (0.97–2.83)	15 (60.0)	1.99 (0.80–4.57)
Social Level (peer norms)							
Friends always use a condom	Yes	217 (36.6)	1 (Ref)	121 (33.8)	1 (Ref)	96 (40.9)	1 (Ref)
with a new partner	No	141 (47.6)	**1.58 (1.19**–**2.09)**	81 (43.1)	**1.48 (1.03–2.13)**	60 (55.6)	**1.81 (1.14–2.87**)
Friends have difficulty	No	159 (36.5)	1 (Ref)	88 (33.3)	1 (Ref)	71 (41.3)	1 (Ref)
demanding condom use with new partner	Yes	207 (44.4)	**1.40 (1.06–1.82)**	118 (40.7)	1.37 (0.97–1.94)	89 (50.6)	1.46 (0.95–2.22)

a Analyzed only for those who consumed alcohol.

Table 3 presents the adjusted OR with 95% CI for association between condom efficacy and inconsistent condom use (adjusted for the confounding factors of sex, age, rural origin, friends who always use a condom with a new partner, friends who have a difficulty demanding a condom, less pleasure using a condom, multiple sex partners, and alcohol consumption in conjunction to sexual intercourse). In the fully-adjusted model, the statistically significant association persisted between low condom efficacy and inconsistent condom use (OR adjusted 3.94, 95% CI 2.20–7.05).

**Table 3:** Association (adjusted odds ratio, 95% CI) between condom efficacy and inconsistent condom use among Ugandan University students

	Model 1	Model 2	Model 3	Model 4	Model 5	Model 6
Low condom efficacy	**4.34 (2.51–7.52)**	**4.03 (3.29–7.08)**	**4.02 (2.28–7.08)**	**3.87 (2.19–6.86)**	**4.00 (2.24–7.11)**	**3.94 (2.20–7.05)**
Female	1.27 (0.79–2.04)	1.29 (0.79–2.10)	1.29 (0.79–2.10)	1.32 (0.80–2.15)	1.25 (0.76–2.07)	1.33 (0.80–2.21)
≤23 years		**1.66 (1.01–2.73)**	**1.66 (1.01–2.73)**	**1.76 (1.07–2.91)**	**1.75 (1.06–2.90)**	**1.79 (1.08–2.97)**
Rural		**2.10 (1.31–3.36)**	**2.09 (1.30–3.35)**	**2.15 (1.33–3.45)**	**2.12 (1.31–3.42)**	**2.18 (1.34–3.54)**
Friends use condom with new partner			1.03 (0.63–1.67)	0.99 (0.60–1.61)	0.98 (0.60–1.61)	0.94 (0.57–1.55)
Friends have difficulty demanding condom use with new partner			1.05 (0.66–1.69)	1.06 (0.66–1.70)	1.08 (0.67–1.75)	1.09 (0.67–1.76)
Less pleasure with using a condom				**1.74 (1.04–2.92)**	**1.75 (1.04–2.93)**	**1.78 (1.06–3.00)**
Multiple sexual partners					0.79 (0.49–1.28)	0.69 (0.41–1.13)
Alcohol in relation to sex						**2.13 (1.19–3.82)**

In Table 4, we formally tested gender as an effect modifier regarding the association between condom efficacy and inconsistent condom use. The result confirmed the possible synergistic effect of gender, i.e. female gender aggravated the impact of low condom efficacy on inconsistent condom use.

**Table 4:** Analysis of effect modification between condom efficacy and gender regarding inconsistent condom use among Ugandan university students (*n*=1,179), presented as adjusted odds ratio (OR) with 95% CI

	*n* (%)	Cases	OR (95% CI)	
High condom efficacy/Male	485 (47.9)	125 (27.7)	1 (ref)	
High condom efficacy/Female	279 (27.5)	279 (27.5)	1.17 (0.84–1.63)	
Low condom efficacy/Male	138 (13.6)	138 (13.6)	**4.66 (3.08–7.07)**	
Low condom efficacy/Female	111 (11.0)	111 (11.0)	**7.55 (4.62–12.33)**	**Synergy index=1.71**
Missing	166			

## Discussion

Our study found inconsistent condom use to be more prevalent among females as compared to males. This may partially be explained by the lower prevalence of condom efficacy, an important determinant of consistent condom use among females. Moreover, the impact of low condom efficacy on inconsistent condom use was considerably higher among females, compared with the impact among males. All this suggests that condom efficacy is an independent determinant for consistent condom use among both males and females, but to a higher degree among females. This could be a significant factor behind the feminization of the HIV/AIDS epidemic in the study setting. The results also show that frequency of alcohol consumption in relation to sexual intercourse was a mediating variable between condom efficacy and inconsistent condom use, but no particular gender differences were observed regarding this determinant.

Young people in Tanzania showed gender differences regarding the predictors of condom use and its association with condom efficacy (30). For males, condom use depended on perceived self-efficacy, perceived self-efficacy for condom use with a long-term partner and having discussed condom use among friends. The predictors for females were discussing condom use with a sex partner and the perceived self-efficacy to refuse sex if the sex partner does not wish to use a condom (30). Therefore, it is evident that different factors effect condom use among men and women, and among them gender power relations do make a difference in condom use negotiation by women. This can also be supported by previous studies conducted on 18–49 year-old women in South Africa and Botswana (31) and on young people in Angola (32). A possible explanation for the gender differences regarding inconsistent condom use might be that condom use may be equated with lack of trust by men, leading to a fear of rejection on the part of women, which might result in non-use of condom (31). This in turn may expose women to the risk of unwanted pregnancies and STIs, including HIV.

Along with condom efficacy, other individual factors, such as reduced pleasure in sexual intercourse with a condom, showed a significant association with inconsistent condom use. This determinant was more common among men in our study. Our finding is supported by a meta-analysis, which shows that reduced pleasure is a robust predictor of non-use of condoms, where gender differences were observed, men reporting that using condoms reduces pleasure had a higher likelihood of non-use (33). There is evidence from previous research that explains the experience of sexual pleasure as a subjective reflection of a complex interplay of emotions, tactile sensations, and cognition, which limits its use among young people (34, 35). However, the difference between males and females in our study was not enough to make it a major explanatory factor for inconsistent condom use, especially since the impact on this behavior was of the same magnitude for males and females.

We found that *intention* to use a condom did not show a significant association with inconsistent condom use. This may be explained by the theory of planned behavior, which posits that behavioral intentions are determined by attitudes, subjective norms, and perceived control, leading to considerable variation in actual behavior under different circumstances (18, 19). Empirical evidence from studies of high school and university students in South Africa similarly found that intention to use a condom is determined by normative beliefs, attitudes, and subjective norms (17, 26). Gender differences with regard to intention have been observed among university students in a study that showed attitude as a better predictor of intention for young women, whereas men rely on subjective norms and their perception of communication and persuasion skills (36). Thus, behavioral intentions may depend more on individual factors for women, while for men social factors are more decisive (36). It appears that merely having the intention to use a condom may not translate into behavior, especially in a Ugandan context where attitudes and socio-cultural norms exert a strong influence.

In our study, alcohol consumption in relation to sex was found to mediate the association between low condom efficacy and inconsistent condom use with a new sex partner. University students who engage in risky alcohol consumption may thereby limit their ability to use a condom. As explained by alcohol myopia theory (37), a person who consumes alcohol experiences a restriction of their cognitive capacity. Such an individual focuses on salient situational cues of sexual initiation and ignores peripheral ones, making them less likely to identify potential dangers, like the risk of an unintended pregnancy or STIs. The consumption of alcohol on the occasion of sexual intercourse was infrequent in our sample, and the impact on inconsistent condom use was relatively similar in men and women. Thus, this factor did not seem to contribute to the observed gender differences in inconsistent condom use that we found.

### Strengths and limitations

One of the strengths of our study was that we addressed gender differences at the individual and social level in relation to condom efficacy and inconsistent condom use. This has not been well investigated previously in a Ugandan university population. A limitation of our study was the cross-sectional study design, as a result of which we could not judge the causal direction. According to our calculations of statistical power, the sample size was adequate for the main analyses, although somewhat small for assessing synergy, but no formal test of statistical significance was made for those analyses. Our relatively high response rate (72%) leaves room for a selection bias in our study. However, the reasons for non-participation do not seem to be linked to the main exposures or to the outcome, but were mostly caused by logistical circumstances. To reduce response bias in our study, the anonymity of the respondents was assured, which may have increased truthful reporting.

Another limitation of the study could be that sensitive questions regarding sexual behaviors might have been underreported, due to the issue of social desirability. If this was the case we believe it would bias the results towards the null, since it would more likely represent a case of non-differential misclassification than differential misclassification. In addition, our study results might not be fully generalizable to all countries, but we feel they may apply to university students in similar settings. In our analysis, we adjusted for the potential confounding factors, and therefore we believe that residual confounding would be of minor importance. Since our assessment of the exposure of condom efficacy was limited to two items, it is possible that if more items were added to the questionnaire we might have had greater disclosure on the efficaciousness of the participants.

## Conclusion

We found that low condom efficacy had an association with inconsistent condom use among male and female university students in Uganda. Females with lower condom efficacy were at a higher risk of inconsistent condom use compared to males. These findings have implications for policy formulation of young people's sexual and reproductive health in Uganda. Gender differences need to be taken into account in order to gain a deeper understanding of the factors that influence condom use in this region. With such knowledge, we can design and implement effective interventions against the spread of HIV/AIDS. It is imperative to acknowledge gender aspects when working towards improving condom efficacy of young people. The issue of gender equality should be addressed when designing intervention strategies that focus on sex education and counseling. These programs should aim at improving interpersonal communication that includes building condom use negotiation skills between partners.
